# Optimization of 4-1BB antibody for cancer immunotherapy by balancing agonistic strength with FcγR affinity

**DOI:** 10.1038/s41467-019-10088-1

**Published:** 2019-05-20

**Authors:** Xinyue Qi, Fanlin Li, Yi Wu, Chen Cheng, Ping Han, Jieyi Wang, Xuanming Yang

**Affiliations:** 1 Sheng Yushou Center of Cell Biology and Immunology, School of Life Sciences and Biotechnology, Shanghai Jiao Tong University, 200240 Shanghai, China; 2 Joint International Research Laboratory of Metabolic & Developmental Sciences, Shanghai Jiao Tong University, 200240 Shanghai, China; 3 Lyvgen Biopharma, 201203 Shanghai, China; 4 Key Laboratory of Systems Biomedicine (Ministry of Education), Shanghai Center for Systems Biomedicine, Shanghai Jiao Tong University, 200240 Shanghai, China

**Keywords:** Antibody therapy, Cancer immunotherapy, Toxicology, Immunotherapy, Tumour immunology

## Abstract

Costimulation of T cell responses with monoclonal antibody agonists (mAb-AG) targeting 4-1BB showed robust anti-tumor activity in preclinical models, but their clinical development was hampered by low efficacy (Utomilumab) or severe liver toxicity (Urelumab). Here we show that isotype and intrinsic agonistic strength co-determine the efficacy and toxicity of anti-4-1BB mAb-AG. While intrinsically strong agonistic anti-4-1BB can activate 4-1BB in the absence of FcγRs, weak agonistic antibodies rely on FcγRs to activate 4-1BB. All FcγRs can crosslink anti-41BB antibodies to strengthen co-stimulation, but activating FcγR-induced antibody-dependent cell-mediated cytotoxicity compromises anti-tumor immunity by deleting 4-1BB^+^ cells. This suggests balancing agonistic activity with the strength of FcγR interaction as a strategy to engineer 4-1BB mAb-AG with optimal therapeutic performance. As a proof of this concept, we have developed LVGN6051, a humanized 4-1BB mAb-AG that shows high anti-tumor efficacy in the absence of liver toxicity in a mouse model of cancer immunotherapy.

## Abstract (web summary)

Agonistic 4-1BB antibodies developed for cancer immunotherapy have suffered from either hepatotoxicity or insufficient anti-cancer activity. Here the authors determine the contribution of FcγR binding and agonistic strength to these outcomes, and engineer a 4-1BB antibody with potent anti-tumor effect and no liver toxicity in mice.

## Introduction

Immune checkpoint blockade antibodies have gained great success in clinic, which aim to release the brake of anti-tumor T cell response. These treatments are effective for only about 30% of patients due to various primary or acquired resistance mechanisms in remaining population. Deep sequencing data have revealed that there are multiple mutations in tumor cells, which are important for tumor development from normal cells. At the same time, these mutations have provided numerous ‘non-self’ targets for immune system recognition, which is the theoretic cornerstone of cancer immunotherapy. Among all the anti-tumor immune responses, T cell-mediated cytotoxic tumor killing is the key for tumor control. Besides first signal through MHC–peptide–TCR axis, both co-inhibitory and co-stimulatory pathways are critical regulators for T cell activation^1^. Therefore, agonistic antibodies (Abs) to co-stimulatory molecules and blocking Abs to co-inhibitory molecules are attractive candidates for cancer immunotherapy. While blocking Abs against PD-L1, PD-1, and CTLA-4 have gained great success^2^, clinical development of agonistic Abs against co-stimulation pathways has significantly lagged behind. Currently, there are five immune check-point blockade Abs approved for cancer therapy. In contrast, there is no agonistic antibody against co-stimulation receptor approved in clinic.

4-1BB, as one representative TNF receptor family co-stimulatory receptor, is expressed on a wide variety of cell types^3,4^, including activated T cells^5^, NK cells^6^, DCs^7^, B cells^8^, monocytes^9^, and neutrophils^10^. 4-1BBL–4-1BB interaction can trigger an activation signal in all these cell types. However, anti-4-1BB-induced CD8^+^ T responses were thought to play a dominant role in anti-tumor immunity^11^. Anti-4-1BB agonistic Abs could induce more effector molecules released from CD8^+^ T cells, increase proliferation and decrease apoptosis of CD8^+^ T cells, which all count for the enhanced anti-tumor immunity^3,12^. Despite better or equivalent anti-tumor activity in preclinical models compared with anti-PD-1 and anti-PD-L1 Abs^11^, two anti-4-1BB Abs entered clinical trials, Urelumab and Utomilumab, remained in early stages. These Abs face different challenges in the clinic: while safe, Utomilumab has relatively low efficacy^13^, and Urelumab causes severe liver toxicity despite anti-tumor efficacy^14^. To achieve optimal therapeutic potential, a deeper understanding of the mechanisms behind the anti-tumor and liver toxicity effects of anti-4-1BB Abs is warranted.

The major function of an Ab is mainly determined by Fab moiety, which contributes to target specificity. However, recently publications have highlighted that the Fc portion also plays important role in regulating Ab’s function through Fc–FcγR interaction^15–18^. There are four FcγRs (FcγRI, FcγRIIB, FcγRIII, and FcγRIV) in mouse and six FcγRs (FcγRI, FcγRIIA, FcγRIIB, FcγRIIC, FcγRIIIA, and FcγRIIIB) in human^19^. Among these FcγRs, FcγRIIB in both mouse and human is the only inhibitory FcγR to transduce suppressive signal via intracellular ITIM motif. Other FcγRs are activating FcγRs and transduce activation signals, such as releasing pro-inflammatory cytokines and promoting ADCC effect, through ITAM motif^19^. It has been well known that activating FcγRs-mediated ADCC and CDC is required for the efficacy of anti-oncogenic receptor Abs. In this scenario, human IgG1 is commonly chosen to achieve maximum ADCC/CDC effect, such as Rituximab, Cetuximab, and Herceptin^20,21^. Recent studies have demonstrated other mechanisms of Fc–FcγR interaction in regulating the efficacy of immune-modulating Abs. For anti-PD-L1 Abs, mIgG2a or hIgG1 is preferred as activating FcγR-mediated depletion of PD-L1^+^ immune suppressive cells contributes to its anti-tumor efficacy^17,22^. In contrast, for anti-PD-1 Abs, it is better to use mIgG1 or hIgG4 to avoid strong ADCC-mediated depletion of effector CD8^+^ T cells^17^. While for anti-CTLA-4 Abs, hIgG1 isotype is critical since depletion of CTLA-4^+^ Treg cells instead of blocking CTLA-4-mediated suppressive signal is dominant mechanism for anti-tumor effect^23,24^. For Abs targeting immune stimulatory molecules, such as anti-CD40 Abs, it requires inhibitory FcγRIIB-mediated crosslinking for agonistic effect^15,18,25^. These complex mechanisms have highlighted the critical role of Fc–FcγR interaction in modulating therapeutic effect of Abs.

In this study, we have demonstrated, for the first time and to our surprise, that the anti-tumor efficacy and liver toxicity can be separated in natural anti-4-1BB agonist Abs. Further investigations reveal that the intrinsic agonistic strength, isotype, and interaction with FcγRs are critical for anti-4-1BB antibody activity and liver toxicity. Manipulating these characters will open new direction for 4-1BB and other TNFR family Abs design and optimization.

## Results

### Distinct activity and toxicity profiles of anti-4-1BB Abs

**Fig. 1:**
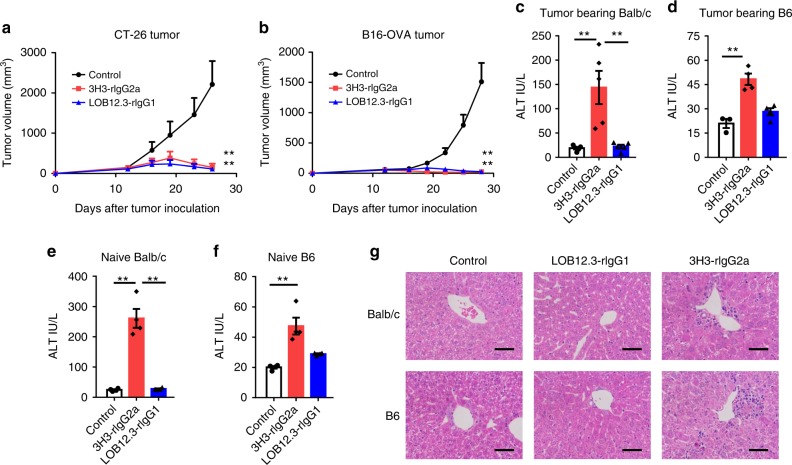
Distinct activity and toxicity profiles of anti-4-1BB Abs. **a** WT Balb/c (*n* = 4–5/group) or **b** B6 mice (*n* = 5/group) were injected subcutaneously with 1 × 10^6^ (**a**) CT-26 (**b**) B16-OVA cells, then 200 μg of anti-4-1BB (3H3 or LOB12.3) or control IgG was administered on days 9, 16, and 23. Tumor growth was measured twice a week. **c**, **d** Twenty-one days after the first treatment, serum ALT levels in **a**, **b** were measured. **e** Naive Balb/c (*n* = 4/group) or **f** B6 mice (*n* = 4/group) were treated with 200 μg of anti-4-1BB (3H3 or LOB12.3) or control IgG on days 0, 7, and 14, and serum ALT levels were measured on day 21. **g** Same as in **e**, **f** liver tissue was stained by HE. Mean ± SEM are shown. *p < 0.05, **p < 0.01 compared with control group or as indicated. Scale bar: 50 µm

To investigate liver toxicity and anti-tumor efficacy of anti-4-1BB Abs in preclinical mouse models, we first screened commercially available rat anti-murine 4-1BB Abs across different tumor types (melanoma B16-OVA and colorectal cancer CT-26) and mouse strains (B6 and Balb/c). In this B16-OVA cell line, OVA is stably expressed and serves as a tumor-specific model antigen. We use B16-OVA model to mimic highly immunogenic cancer in clinic. Both LOB12.3 and 3H3 Abs showed anti-tumor efficacy (Fig. 1a, b) but they exhibited distinct liver toxicity profiles; 3H3 significantly increased alanine transaminase (ALT) levels, whereas LOB12.3 had minimal impact on ALT levels (Fig. 1c, d). We observed similarly increased ALT levels in 3H3-treated naive mice (Fig. 1e, f), suggesting 3H3-induced liver toxicity is independent of tumor burden. Besides elevation of ALT in serum, we also found that immune cell infiltration in the liver of 3H3-treated mice (Fig. 1g). Our data suggested anti-4-1BB Ab-induced anti-tumor activity and liver toxicity could be separated in natural anti-4-1BB Abs. We, therefore, used LOB12.3 and 3H3 Abs as tool molecules to investigate the underlying mechanism in order to gain insights for clinical development of agonistic anti-4-1BB Abs.

### FcγRs-crosslinking enhanced the activity of anti-4-1BB Abs

**Fig. 2:**
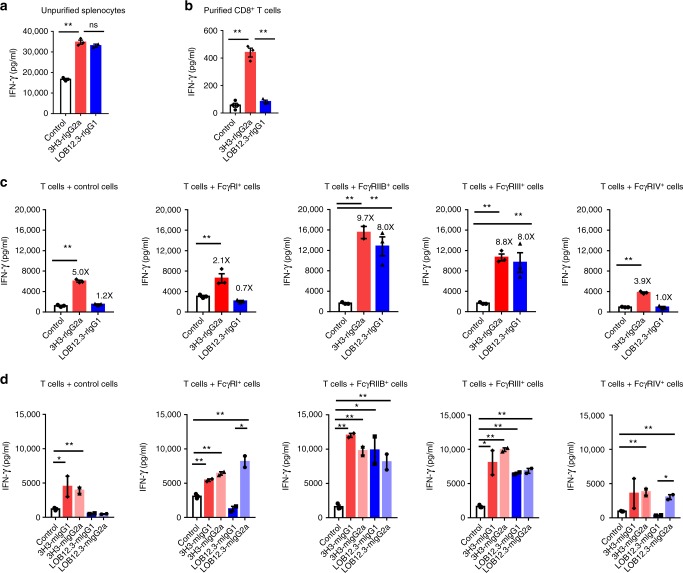
FcγRs-crosslinking enhanced the activity of anti-4-1BB Abs. **a** Total splenocytes or **b** purified CD8^+^ T cells from B6 mice were stimulated with anti-CD3 and indicated anti-4-1BB Abs, 2 days later, the IFN-γ in culture medium was analyzed by CBA. **c**, **d** Purified CD8^+^ T cells from B6 mice were stimulated with anti-CD3 and indicated anti-4-1BB Abs when indicated single mouse FcγR-expressing B16-OVA cells were present, 2 days later, the IFN-γ in culture medium was analyzed by CBA. Mean ± SEM are shown. *p < 0.05, **p < 0.01 compared with control group or as indicated

Since 4-1BB is a critical T cell co-stimulatory receptor^26^, we analyzed whether the distinct anti-tumor and liver toxicity profiles of 3H3 and LOB12.3 were due to differences in their ability to activate T cells. Using an in vitro co-stimulation assay, we found LOB12.3 and 3H3 had a comparable ability to activate T cells in total splenocyte preparations (Fig. 2a). To test whether T cell activation was dependent on help from other immune cells, we replaced total splenocytes with purified CD8^+^ T cells in the co-stimulation assay. Surprisingly, only 3H3 showed a co-stimulatory effect, suggesting the co-stimulatory ability of 3H3 does not depend on non-T cells while that of LOB12.3 requires help from other cells (Fig. 2b). This was not due to affinity differences, as affinity to 4-1BB was comparable (Supplementary Fig. 1A). The IFN-γ production was lower from purified T cells than splenocytes, which may be due to the lack of positive feedback loop between T cells and antigen-presenting cells.

To explore FcγR requirements for co-stimulatory activity of anti-4-1BB Ab, we constructed stable cell lines expressing single mouse FcγRs (Supplementary Fig. 2) to mimic FcγR from non-T cells. In co-cultures of FcγRIIB-expressing or FcγRIII-expressing cells and purified CD8^+^ T cells, LOB12.3’s co-stimulatory activity was restored (Fig. 2c). This was consistent with the finding that LOB12.3’s isotype (rat IgG1) could only bind to mouse FcγRIIB and FcγRIII (Supplementary Fig. 1B). The co-stimulatory activity of 3H3 was consistently high whether FcγR-expressing cells were present or not although FcγR-expressing cells enhanced the activity (Fig. 2c). To determine the roles of specific Fc, we engineered chimeric mouse IgG1/IgG2a LOB12.3 and 3H3, while avoiding cross-species immune responses. By using the same in vitro T cell co-stimulation assay, LOB12.3-mIgG1 showed strong co-stimulation ability when FcγRIIB or FcγRIII was present (Fig. 2d). While for LOB12.3-mIgG2a Abs, all FcγRs could crosslink and promote its co-stimulation ability (Fig. 2d). When FcγRIIB or III was present, the agonistic activity of 3H3 mIgG1/mIgG2a was further enhanced (Fig. 2d). These data suggested that agonistic activity of anti-4-1BB Abs varies, which could be further enhanced by FcγRs-mediated crosslinking. Aggregated Abs can perform a crosslinking-like function to promote agonistic effect. To test whether antibody aggregation could contribute to the strong agonistic activity of 3H3-derived Abs, we performed size-exclusion chromatography (SEC) analysis and there was no antibody aggregation observed from 3H3-derived Abs (3H3-rIgG1, 3H3-mIgG1, and 3H3-mIgG2a) (Supplementary Fig. 3). It suggests that the strong agonistic feature of 3H3-derived Abs is not due to antibody aggregation. Based on these results, we proposed the concept of strong agonistic or weak agonistic anti-4-1BB Ab: strong agonistic anti-4-1BB Ab could activate 4-1BB without the crosslinking from FcγR engagement, while weak agonistic anti-4-1BB requires FcγR-mediated crosslinking to activate 4-1BB signaling. Both weak and strong agonistic anti-4-1BB Abs could be further enhanced by FcγR-mediated crosslinking.

### Activating FcγRs compromise anti-4-1BB Abs’ activity in vivo

Consistent with potent T cell activation in vitro, 3H3-rIgG2a and LOB12.3-rIgG1 treatment induced strong tumor-specific effector CD8^+^ T cell responses (Supplementary Fig. 4A). Importantly, CD8^+^ T cell depletion eliminated the therapeutic effect of anti-4-1BB Abs, suggesting anti-4-1BB-induced CTL responses are essential for their anti-tumor effect (Supplementary Fig. 4B–E). To test whether anti-4-1BB treatment could generate anti-tumor memory responses, we challenged cured mice with lethal dosage of CT-26 tumor cells. Both 3H3-rIgG2a and LOB12.3-rIgG1 showed a protective effect, suggesting an existing tumor-specific memory response (Supplementary Fig. 4F).

**Fig. 3:**
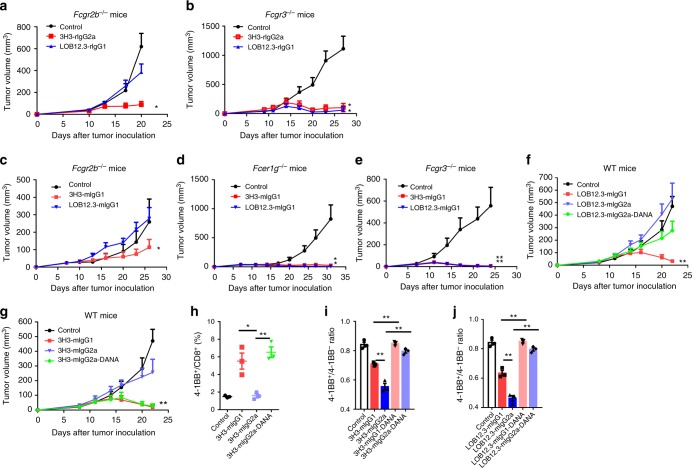
Activating FcγRs compromise anti-4-1BB Abs’ activity in vivo. **a** *Fcgr2b*^−/−^ (*n* = 6–9/group) and **b** *Fcgr3*^−/−^ mice (*n* = 5/group) were injected subcutaneously with 1 × 10^6^ B16-OVA cells, then 200 μg of indicated control IgG or anti-4-1BB Abs were administered on days 9, 16, and 23. Tumor growth was measured twice a week. **c**–**e** *Fcgr2b*^−/−^ (*n* = 6–7/group), *Fcer1g*^−/−^ (*n* = 7/group) and *Fcgr3*^−/−^ mice (*n* = 5–6/group) were injected subcutaneously with 1 × 10^6^ B16-OVA cells, then 200 μg of indicated control IgG or chimeric anti-4-1BB Abs were administered on days 9, 16, and 23. Tumor growth was measured twice a week. **f**, **g** WT Balb/c mice (*n* = 5/group) were injected subcutaneously with 1 × 10^6^ CT-26 cells, then 200 μg of indicated chimeric anti-4-1BB Abs or control IgG was administered on days 9, 16, and 23. Tumor growth was measured twice a week. **h** Same as in **g**, splenocytes were analyzed by flow cytometry. **i**, **j** Splenocytes from *Rag1*^−/−^ mice were co-cultured with EL4-4-1BB^+^ CFSE^high^ and EL4-4-1BB^-^ CFSE^low^ cells in the presence of indicated anti-4-1BB Abs. One day later, the ratio of EL4-4-1BB^+^/4-1BB^−^ was analyzed by flow cytometry. Mean ± SEM are shown. *p < 0.05, **p < 0.01 compared with control group or as indicated

Our in vitro data indicated both FcγRIIB and FcγRIII can crosslink LOB12.3-rIgG1 to activate 4-1BB co-stimulation pathway on T cells; we next explored whether FcγRIIB and FcγRIII are required for anti-tumor activity in vivo. In contrast to in vitro observations, anti-tumor efficacy of LOB12.3-rIgG1 was significantly impaired in *Fcgr2b*^−/−^ but not *Fcgr3*^−/−^ mice (Fig. 3a, b). Consistent with its strong co-stimulation activity in vitro, 3H3-rIgG2a showed anti-tumor activity in both *Fcgr2b*^−/−^ and *Fcgr3*^−/−^ mice (Fig. 3a, b). To avoid cross-species immune responses, we performed the tumor experiments with 3H3 and LOB12.3 with mouse IgG1 isotype since mIgG1 has similar mouse FcγRs-binding profile as parent rat IgG1 and rat IgG2a. We observed similar phenotype that the anti-tumor activity of LOB12.3-mIgG1 is significantly reduced in *Fcgr2b*^−/−^, while 3H3-mIgG1 showed a weak but significant anti-tumor activity in *Fcgr2b*^−/−^ mice (Fig. 3c). In *Fcer1g*^−/−^ and *Fcgr3*^−/−^ mice, LOB12.3-mIgG1 and 3H3-mIgG1 showed potent anti-tumor activity similar as in WT mice (Fig. 3d, e). These data suggested that mouse immune response to rat isotypes is not the cause in this scenario. Taken together, these data indicate that FcγRIIB is required for anti-tumor activity of weak agonistic anti-4-1BB Abs in vivo.

Since ADCC is a major event following engagement of activating FcγRs^19^, we hypothesized that downstream signaling of activating FcγRs could induce ADCC to delete 4-1BB^+^ effector cells. To test this idea, we compared the anti-tumor activity of LOB12.3-mIgG1, LOB12.3-mIgG2a, 3H3-mIgG1, and 3H3-mIgG2a. Mouse IgG2a is similar to human IgG1, which is the strongest isotype to induce ADCC in mice. When strong ADCC-inducing isotype mIgG2a is engineered, the anti-tumor activity is significantly reduced for both 3H3 and LOB12.3 (Fig. 3f, g). To test whether anti-tumor activity of 3H3-mIgG2a could be restored by blocking FcγR-mediated ADCC, we generated 3H3-mIgG2a with FcγR-binding null mutations D265A and N297A (DANA), resulting in Abs unable to bind any FcγRs^27^. 3H3-mIgG2a-DANA had enhanced anti-tumor activity compared with 3H3-mIgG2a (Fig. 3g), suggesting a negative role for activating FcγRs and ADCC in anti-4-1BB-mediated anti-tumor responses. Of note, both LOB12.3-mIgG2a and LOB12.3-mIgG2a-DANA had the same weak anti-tumor effect: for LOB12.3-mIgG2a, the low efficacy is due to strong ADCC-mediated depletion by mIgG2a isotype; while for LOB12.3-mIgG2a-DANA, the lower efficacy is due to lack of FcγR-mediated crosslinking. 4-1BB^+^ CD8^+^ T cell numbers were also significantly reduced in 3H3-mIgG2a-treated versus 3H3-mIgG1-treated mice, and were restored with 3H3-mIgG2a-DANA treatment (Fig. 3h, Supplementary Fig. 5A), which is consistent with predicted ADCC-mediated depletion effect. To provide direct evidence of ADCC-induced 4-1BB^+^ cell depletion, we developed an in vitro assay with target cells (4-1BB^+^ and 4-1BB^−^EL4) and innate effector cells from *Rag1*^−/−^ mice. Consistent with in vivo observations, 3H3-mIgG2a induced more killing of 4-1BB^+^ target cells than 3H3-mIgG1, while 3H3-mIgG2a-DANA-induced minimal killing (Fig. 3i, Supplementary Fig. 5B). LOB12.3 Abs showed a similar effect (Fig. 3j). Our data suggest that although activating FcγRs can crosslink and activate 4-1BB, ADCC leads to depletion of 4-1BB^+^ effector cells, compromising anti-tumor activity.

### Fab and Fc co-determine liver toxicity by anti-4-1BB Abs

**Fig. 4:**
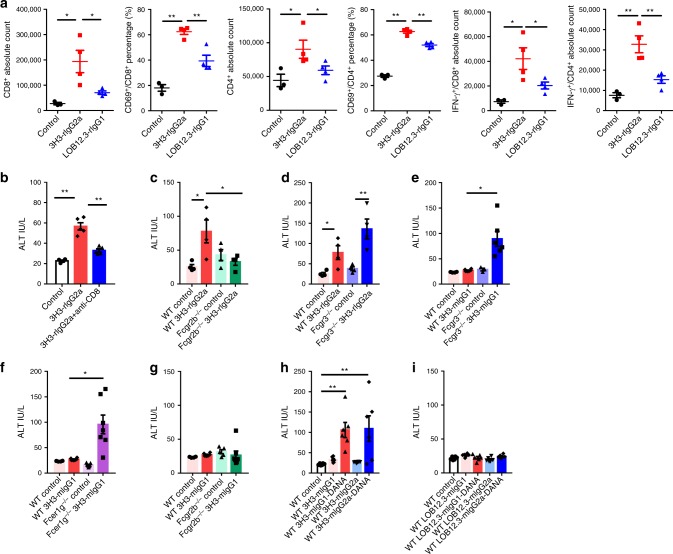
Fab and Fc co-determine liver toxicity by anti-4-1BB Abs. **a** After anti-4-1BB Ab treatment, liver leukocytes (*n* = 3–4/group) were analyzed by flow cytometry directly or stimulated with PMA/Ionomycin/BFA for 1 h, followed by intracellular staining for IFN-γ. **b** WT B6 mice (*n* = 4–5/group) were treated with 200 μg of anti-4-1BB Abs on days 0, 7, and 14, with 200 μg of anti-CD8 administered on days 0 and 7. **c**, **d** *Fcgr2b*^−/−^ (*n* = 4/group) and *Fcgr3*^−/−^ mice (*n* = 4/group) were treated with 200 μg of anti-4-1BB rat Abs on days 0, 7, and 14. **e**–**g** *Fcgr3*^−/−^ (*n* = 3–6/group)*, Fcer1g*^−/−^ (*n* = 4–7/group), and *Fcgr2b*^−/−^ mice (*n* = 4–7/group) were treated with 200 μg of anti-4-1BB chimeric Abs on days 0, 7, and 14. **h**, **i** WT Balb/c mice (*n* = 6/group) were treated with indicated anti-4-1BB chimeric Abs on days 0, 7, and 14. **b**–**i** All serum ALT levels were analyzed on day 21 after first antibody treatment. Mean ± SEM are shown. *p < 0.05, **p < 0.01 compared with control group or as indicated

We next analyzed the cell profile in the liver after anti-4-1BB treatment. Infiltration of CD45^+^ leukocytes and CD4^+^ and CD8^+^ T cells dramatically increased in 3H3-treated, but not in LOB12.3-treated mice (Supplementary Fig. 6A&B). These CD4^+^ and CD8^+^ T cells were activated, with upregulated CD69 expression and IFN-γ production (Fig. 4a, Supplementary Fig. 7). Depletion of CD8^+^ T cells decreased ALT while depletion of CD4^+^ T cells showed no effect (Fig. 4b, Supplementary Fig. 6C), suggesting CD8^+^ T cell activation is critical for 3H3-mediated liver toxicity. To explore whether FcγRs contribute to liver toxicity and potential directions for Ab optimization, we evaluated 3H3-induced liver damage in *Fcgr2b*^−/−^ and *Fcgr3*^−/−^ mice. We found 3H3-mediated ALT elevation and immune cell infiltration were diminished in *Fcgr2b*^−/−^ but slightly increased in *Fcgr3*^−/−^ mice (Fig. 4c, d, Supplementary Fig. 6D&E). To avoid cross-species immune responses, we performed the liver toxicity experiments with 3H3-mouse IgG1 isotype. There is a slightly ALT elevation in 3H3-mIgG1-treated WT mice. However the ALT elevation is significantly increased in *Fcgr3*^−/−^ and *Fcer1g*^−/−^ *mice*, but not in *Fcgr2b*^−/−^ mice (Fig. 4e–g). This suggests binding of the 3H3 Fc to inhibitory FcγRIIB and activating FcγRIII has opposing roles in 3H3-induced liver toxicity, raising the possibility that altering the ratio of activating-to-inhibitory (A/I) FcγRs bound to the Fc can modulate liver toxicity effects. We generated 3H3-mIgG1, 3H3-mIgG2a, 3H3-mIgG1-DANA, and 3H3-mIgG2a-DANA, which represent isotypes with increasing A/I FcγR-binding ratio: (mIgG1-DANA or mIgG2a-DANA)<mIgG1<mIgG2a. There is a slightly ALT elevation in 3H3-mIgG1-treated mice, while 3H3-mIgG1-DANA and 3H3-mIgG2a-DANA induced significant ALT elevation (Fig. 4h), suggesting an isotype with a low A/I FcγR-binding ratio is essential for 3H3-induced liver toxicity. We have performed the same experiment with LOB12.3, there is no ALT elevation for LOB12.3-mIgG1, LOB12.3-mIgG1DANA, LOB12.3-mIgG2a, and LOB12.3-mIgG2aDANA (Fig. 4i). Collectively, both a strong agonistic anti-4-1BB Fab and an Fc with a low A/I binding ratio are indispensable for anti-4-1BB-mediated liver toxicity.

### Activation and toxicity profile of human anti-4-1BB Abs

**Fig. 5:**
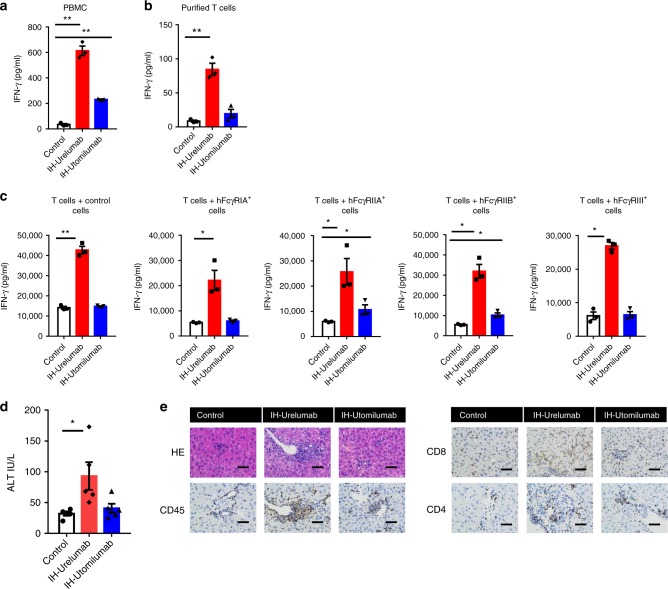
Activation and toxicity profile of human anti-4-1BB Abs. **a** Human PBMC or **b** purified T cells from human PBMC were stimulated with anti-CD3 and indicated anti-4-1BB Abs. Two days later, the IFN-γ in culture medium was analyzed by CBA assay. **c** Purified T cells from h-4-1BB KI B6 mice were stimulated with anti-CD3 and indicated anti-human 4-1BB Abs in the presence of single human FcγR-expressing 3T3 cells. Two days later, the IFN-γ in culture medium was analyzed by CBA assay. **d** Human 4-1BB KI BMC mice (*n* = 5/group) were treated with 200 μg of indicated anti-4-1BB Abs on days 0, 7, and 14. Serum ALT levels were analyzed on day 21. **e** Same as in **d**, liver tissue was stained by HE and IHC. Mean ± SEM are shown. *p < 0.05, **p < 0.01 compared with control group or as indicated. Scale bar: 50 µm

To test whether the characteristics of strong agonistic and weak agonistic anti-mouse 4-1BB Abs were applicable to anti-human 4-1BB Abs, we prepared in-house versions of Urelumab (IH-Urelumab) and Utomilumab (IH-Utomilumab). In PBMC and purified T cell settings, in-house Urelumab was able to co-stimulate T cells in the absence of FcγR (Fig. 5a, b), while in-house Utomilumab required FcγRIIA-expressing or FcγRIIB-expressing cells for its agonistic activity (Fig. 5c). This suggests Urelumab is a strong agonistic Ab and Utomilumab is a weak one. To understand why Urelumab but not Utomilumab induced liver toxicity in clinical studies^14^, we utilized human 4-1BB transgenic bone marrow chimeric mice to evaluate the liver toxicity of in-house Urelumab and in-house Utomilumab, neither of which recognize murine 4-1BB. Consistent with clinical observations, in-house Urelumab induced significant ALT elevation and immune cell infiltration, whereas in-house Utomilumab did not (Fig. 5d, e). Thus, the MoAs for strong and weak agonistic anti-mouse 4-1BB Abs were also applicable to human anti-4-1BB Abs. Strong agonistic Abs can activate 4-1BB in the absence of FcγRs with potential to induce liver toxicity, whereas weak agonistic Abs rely on FcγR crosslinking for 4-1BB activation, do not induce liver toxicity, and maintain anti-tumor efficacy.

### Engineering potent anti-h4-1BB Ab with limited toxicity

**Fig. 6:**
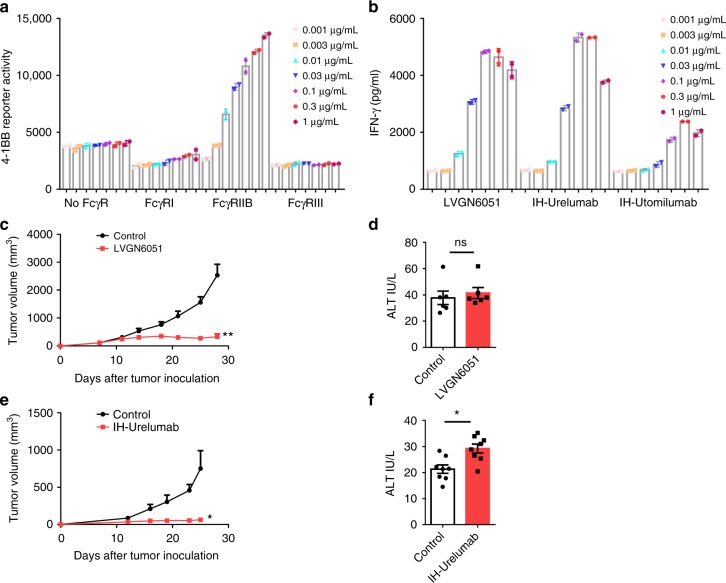
Engineering potent anti-h4-1BB Ab with limited toxicity. **a** GS-H2/4-1BB reporter cell line was stimulated with LVGN6051 anti-4-1BB Ab in the presence of single human FcγR-expressing cells. Two days later, reporter activity was analyzed. **b** Purified T cells from human PBMC were stimulated with anti-CD3 and indicated anti-4-1BB Abs in the presence of human FcγRIIB-expressing cells. Two days later, the IFN-γ in culture medium was analyzed by CBA assay. **c** Human 4-1BB KI mice (*n* = 5–6/group) were treated with 200 μg of anti-4-1BB Ab LVGN6051. Tumor growth was measured twice a week. **d** Same as in **c**, Serum ALT levels were analyzed on day 21. **e** Human 4-1BB KI mice (*n* = 7/group) were treated with 200 μg of in-house Urelumab. Tumor growth was measured twice a week. **f** Same as in **e**, Serum ALT levels were analyzed on day 21 after first antibody treatment. Mean± SEM are shown. *p < 0.05, **p < 0.01 compared with control group or as indicated

Based on our data with both anti-m4-1BB and anit-h4-1BB Abs, we proposed that a weak agonistic Fab plus an engineered Fc with selective FcγRIIB binding might show good efficacy and safety profile. To test our hypothesis, we have engineered a novel anti-human-41BB Ab LVGN6051 to meet these criteria. It is a weak agonistic Ab which required FcγRIIB-mediated crosslinking for agonistic activity. In 4-1BB activation reporter assay, LVGN6051 showed significantly improved co-stimulation ability when FcγRIIB is present (Fig. 6a). Then we compared the T cell co-stimulation ability of LVGN6051, in-house Urelumab, and in-house Utomilumab. Due to its FcγRIIB selective Fc, LVGN6051 showed T cell co-stimulation ability comparable to in-house Urelumab and superior to in-house Utomilumab when FcγRIIB-expressing cells are present (Fig. 6b). Furthermore, LVGN6051 showed robust tumor control ability in a wide range of dosage (Fig. 6c, Supplementary Fig. 8A). Most importantly, it did not induce liver toxicity while maintained potent anti-tumor activity (Fig. 6d, Supplementary Fig. 8B). In similar condition, in-house Urelumab showed potent anti-tumor activity (Fig. 6e). However, it also induced significant liver toxicity (Fig. 6f). This proof-of-concept study demonstrated that our strategy can practically direct the screen and optimization of clinical anti-4-1BB therapeutic candidates.

## Discussion

**Fig. 7:**
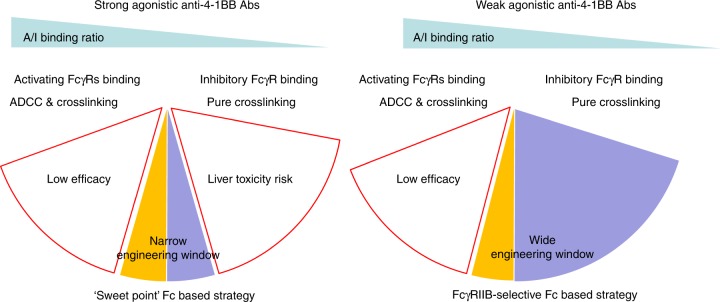
Optimization strategies for different anti-4-1BB Abs. Strong agonistic anti-4-1BB Abs can co-stimulate T cell when FcγRs are absent. Weak agonistic anti-4-1BB Abs require FcγR-mediated crosslinking for activity. When anti-4-1BB Abs are crosslinked by activating FcγRs, ADCC will comprise the co-stimulation ability. When anti-4-1BB Abs are crosslinked by FcγRIIB, it will not cause ADCC and induce strong T cell activation. When strong agonistic anti-4-1BB are engineered with low A/I Fc, it can cause super-activation of T cells and induce liver toxicity. For optimization of strong agonistic anti-4-1BB Abs, it requires fine tuning the Fc within a narrow range to balancing efficacy and toxicity. For optimization of weak agonistic anti-4-1BB Abs, if equipped with FcγRIIB selective Fc, it shows potent anti-tumor activity with desired liver safety profile

Co-stimulation receptor agonistic Abs are important cancer therapeutic candidates. Among them, anti-4-1BB Abs have shown promising anti-tumor effect in preclinical models. However, its clinical development is facing serious challenges due to low efficacy or severe liver toxicity. In this study, we demonstrate for the first time that the anti-tumor efficacy and liver toxicity characteristics of anti-4-1BB agonist Abs can be separated based on the agonistic ability and isotype. The combination of intrinsic agonistic strength and Fc determine the anti-tumor and liver toxicity property of anti-4-1BB agonist Abs. Anti-4-1BB Fab regions, classified as either strong intrinsic agonistic or weak agonistic Abs, can activate 4-1BB signaling independent of or dependent on FcγR crosslinking, respectively. The contribution of the Fc to anti-4-1BB Ab activity is two-fold. First, all FcγRs can crosslink anti-41BB Fc to induce 4-1BB activation. Second, the nature of the FcγR binding the Fc determines whether ADCC occurs. Activating FcγR binding can mediate ADCC, deleting 4-1BB^+^ effector cells modulate liver toxicity and compromising anti-tumor efficacy, while inhibitory FcγRs cannot induce ADCC and are purely a crosslinking and activation scaffold (Fig. 7).

Although immune checkpoint blockade Abs have shown promising efficacy in some types of cancer, its overall responding rate is still low, about 30%^2,28^. The aim of immune checkpoint blockade is to restore the suppressed anti-tumor immunity to normal level. A lot of intrinsic mechanisms, especially tumor mutation burden which is highlighted by recent publications, have limited the endogenous anti-tumor T cell responses^29^. Agonist Abs against co-stimulatory receptors in T cells or TNFR in APCs are good choices for enhancing immune response in these scenarios. Despite significant interest and effort on these targets, the fact is that none of them has entered into drug market. One limitation factor is liver toxicity, which is a common side effect for most agonistic TNFR Abs^30–34^. It largely compromises the safety window as well as anti-tumor efficacy. Based on our data, FcγRIIB-mediated CD8^+^ T cell activation in the liver contributes to anti-4-1BB-mediated liver toxicity. Since FcγRIIB is expressed on liver sinusoidal endothelial cells and Kupffer cells^35^, we hypothesize that the highly enriched FcγRIIB environment may provide super crosslinking and activation condition for agonistic anti-4-1BB Abs to induce liver toxicity. It will be interesting to explore whether this can be applied to other TNFR family agonistic Abs and the mechanism of differential anti-4-1BB Ab activity in liver and tumor microenvironments. One possible explanation is that the threshold for T cell activation is different in the liver and tumor. In the liver, most T cells are naive cells and there is no strong first signal for T cell activation. Therefore, it requires strong co-stimulation to activate T cell in the liver, such as strong agonistic anti-4-1BB Ab with low A/I ratio of Fc. In contrast, in the tumor microenvironment, the first signal for T cell activation is stronger and the requirement for co-stimulation signal is relatively lower. Therefore, a weak agonistic Ab with proper crosslinking is sufficient to activate T cells in the tumor but not strong enough to activate T cells in the liver.

Based on our observation, we subgroup the agonistic anti-4-1BB Abs to strong agonistic Ab and weak agonistic Ab. These two kinds of Abs are not only present in our preclinical research but also exist in clinical usage, indicating the theoretic and practical importance of this definition. The strong agonistic Abs (3H3 and Urelumab) can activate 4-1BB without FcγR-mediated crosslinking, while the weak agonistic Abs (LOB12.3 and Utomilumab) require FcγR-mediated crosslinking to activate 4-1BB. The intrinsic mechanism of agonistic strength of different anti-4-1BB Abs remains unclear. Recent research has highlighted the binding epitope is important for the agonistic strength of anti-CD40 Abs^36^. It will be interesting to investigate whether similar mechanism could be applied to anti-4-1BB Abs. Besides that, the flexibility/rigidity of antibody may contribute to the agonistic strength. Fine crystal structure of antibody-4-1BB complex will provide insights for the underlying mechanism. Previous research has revealed that FcγRIIB-mediated crosslinking is required for the activity of anti-CD40 and anti-DR5 Abs which are likely to be weak agonistic Abs^15,37^. Considering the similar activation fashion of TNFR family members, this strong/weak agonistic Ab concept could potentially apply to other TNFR members. It is important to determine the nature of an antibody belongs to strong or weak agonistic Ab at the first place and then investigate the role of Fc in regulating the activity of Abs. Furthermore, our findings could open a new direction for TNFR agonistic Ab screening. Most agonistic Abs against TNFR family members are screened through cell line-based assays. If FcγRs are not present in these assays, the weak agonistic antibody will be excluded. Our results show that the weak agonistic Abs exhibit similar anti-tumor efficacy as strong agonistic Abs, while their liver toxicity is minimal. Including FcγRs in the primary screening assay will provide weak agonistic Ab candidates for further optimization. Overall, the concept of strong and weak agonistic Abs will greatly clarify the research field of agonistic Abs against TNFR family members and provide new strategy for agonistic Ab screening.

Recent publications have highlighted the important role of Fc in regulating the activity of cancer therapeutic Abs. Therefore, engineering Fc to desired FcγR-binding profile provides a valuable tool for Ab optimization. It is reported that the anti-tumor efficacy of anti-CD40 can be further enhanced by engineering the Fc to have a low A/I FcγR binding ratio^18^. Here we propose a modified strategy based on our findings with different categories of anti-4-1BB Abs. For a strong agonistic Fab, an isotype with a low A/I FcγR-binding ratio can reduce ADCC to improve anti-tumor activity, but may also cause severe liver toxicity as observed with Urelumab, 2A, or 3H3 (Fig. 7). The Fc selection for strong agonistic anti-4-1BB Abs needs to be fine titrated to achieve the balance of crosslinking and depletion. For a weak agonistic Fab, an isotype with a low A/I FcγR-binding ratio (ideally selective binding to FcγRIIB) can have strong anti-tumor activity without liver toxicity (Fig. 7). Thus with proper cataloging, fine-tuning the A/I-binding ratio through engineering Fc can provide a powerful tool for anti-4-1BB Ab optimization.

Crosslinking by FcγRIIB has been shown to play a unique role for the activity of anti-CD40 Abs. Here we present two separated mechanism to explain the role of Fc in regulating the activity of anti-4-1BB Abs. First, all the FcγRs, not limited to FcγRIIB, could provide crosslinking function for agonistic activity. For example, weak agonistic anti-mouse 4-1BB Abs with mIgG2a isotype could be crosslinked and activated by any mouse FcγR-expressing cell lines in vitro (Fig. 2d). While for the same Fabs with mIgG1 isotype, they can only be crosslinked by mFcγRIIB /III. Second, if FcγR harbors ITAM motif, the FcγR^+^ cells will delete the 4-1BB^+^ cells through ADCC effect, which will compromise the anti-tumor activity of anti-4-1BB Abs. If FcγR harbors ITIM motif, the FcγR^+^ cells will only perform a pure crosslinking platform and induce strong activation of 4-1BB signal pathway.

Collectively, the concept of strong agonistic and weak agonistic anti-4-1BB Abs may be applicable to other TNFR family member Abs, or more broadly, receptor agonistic Abs. Proper categorization of these Abs, will further clarify the mechanisms behind therapeutic activity and accelerate clinical agonistic antibody development. Future studies will focus on optimizing anti-h4-1BB agonist Abs for effective tumor control without liver toxicity.

## Methods

### Mice

C57BL/6J and Balb/c mice were purchased from Beijing Vital River Laboratory Animal Technology Co., Ltd. (Beijing, China). *Fcgr2b*^−/−^ and *Fcgr3*^−/−^ mice were purchased from JAX. Human 4-1BB knock-in mice were purchased from Biocytogen, Inc. (Beijing, China). The human 4-1BB extracellular domain is knocked in and replaced with mouse 4-1BB extracellular domain. All mice were maintained under specific pathogen-free conditions. Animal care and use were in accordance with institutional and NIH protocols and guidelines, and all studies were approved by the Animal Care and Use Committee of Shanghai Jiao Tong University.

### Cell lines and reagents

B16-OVA was kindly provided by Hans Schreiber (The University of Chicago). Lenti-X 293 was purchased from Clontech. 3T3 and CT-26 were kindly provided by Stem Cell Bank, Chinese Academy of Sciences (Shanghai, China) . GS-H2-4-1BB is an engineered reporter cell line stably expressing human 4-1BB receptor whose activation leads to IL-8 production (Genscript). To establish mouse FcγRs-expressing B16-OVA cell lines, B16-OVA were infected with mouse FcγR-expressing lentivirus. After selection with puromycin, pooled resistant cells were identified by anti-FcγR flow cytometry analysis. To establish human FcγR-expressing 3T3 cell lines, 3T3 were infected with human FcγR-expressing lentivirus. After selection with puromycin, pooled resistant cells were identified by anti-FcγR flow cytometry. Human PBMCs from cord blood were kindly provided by Shanghai Longyao Biotechnology Co., Ltd. (Shanghai, China). Informed written consent was obtained from all study participants and the protocol was approved by the Suqian Obstetrics and Gynecology Hospital Ethics Committee (Jiangsu, China). B16-OVA, Lenti-X 293, 3T3, and their derivatives were cultured in 5% CO_2_ and maintained in vitro in DMEM supplemented with 10% heat-inactivated fetal bovine serum (Gibco), 2 mmol/L l-glutamine, 100 units/mL penicillin, and 100 μg/mL streptomycin. Anti-CD8 (YTS 169.4.2) and anti-CD4 (GK1.5) Abs were produced in-house. Anti-mouse 4-1BB (3H3 and LOB12.3) Abs were purchased from BioXcell.

### Production of anti-h4-1BB and anti-m4-1BB Abs

For LOB12.3 and 3H3-derived Abs, the VL and VH of LOB12.3 and 3H3 were synthesized according to published amino acid sequence^38^. Using indicated VH and mouse IgG1 and IgG2a constant region as template, the full length of heavy chain was obtained by overlapping PCR. Using indicated VL and mouse kappa constant region as template, the full length of light chain was obtained by overlapping PCR. These constructs are further cloned into pCDH-EF1-MCS vector. The indicated plasmids containing anti-m4-1BB were transfected into Lenti-X 293 (Clontech) cells and supernatants were collected and purified by Protein A column according to the manual (Repligen Corporation).

For the anti-h4-1BB Abs Urelumab and Utomilumab, the LC and HC regions of anti-4-1BB were obtained from the published patent US8137667B2 and US 2012/0237498A1. The sequences were synthesized by Genewiz (Suzhou, China). The in-house Urelumab and Utomilumab were produced by ExpiCHO system (Invitrogen) and purified by Protein A column according to the manual (Repligen Corporation).

To generate anti-human 4-1BB antibody LYGN6051, Balb/C and SJL mice were immunized with human 4-1BB protein and pcDNA3.1-human CD137 plasmid. Standard hybridoma method was used to obtain monoclonal Abs against human 4-1BB using the splenocytes of the immunized mice. The Abs were screened for 4-1BB binding by standard ELISA and FACS protocols using recombinant 4-1BB protein and CHO cells transfected to overexpress 4-1BB. High-affinity-binding Abs were then tested in CD8 T cell co-stimulation assays with and without crosslinking by cellular FcγRIIB. The clone 605 with FcγRIIB-dependent agonistic activity was selected for further engineering. The heavy and light chain variable region genes were cloned by 5′ RACE system. After humanization using CDR grafting, the variable regions were combined to human kappa and engineered human IgG with point mutations to eliminate binding to the activating FcγRs including FcγRI, FcγRIIA, and FcγRIIIA while retain binding to the inhibitory Fc receptor FcγRIIB and FcRn. The resulting 4-1BB agonist antibody LVGN6051 selectively binds human FcγRIIB and with no detectable binding to other human-activating FcγRs. The recombinant antibody LVGN6051 was produced by stable CHO system and purified by Protein A affinity chromatography followed by ion exchange chromatography. The purified Abs were checked for endotoxin (<5 EU/mg) and monomerization (>95%).

### Tumor growth and treatments

Approximately 0.5–1 × 10^6^ B16-OVA or CT-26 cells were injected subcutaneously on the right flank into 5–12-week-old mice. Tumor volumes were measured along three orthogonal axes (*a*, *b*, and *c*) and calculated as tumor volume = *abc*/2. After tumor was established (~9–12 days), mice were treated with intraperitoneal injections of 200 μg anti-4-1BB or control antibody once a week for 3 weeks. For cell-depleting experiments, 200 μg of anti-CD8 or anti-CD4 Abs were injected intraperitoneally at the same time as the anti-4-1BB treatment.

### Measurement of IFN-γ

OT-I or OT-II peptide-reactive T cells were measured by CBA assay. Spleen or lymph node cells were resuspended in RPMI 1640 supplemented with 10% FBS, 2 mmol/L l-glutamine, 100 units/mL penicillin, and 100 μg/mL streptomycin. A total of 1–4 × 10^5^ spleen or lymph node cells were used for the assay. OT-I or OT-II peptide was added at a concentration of 10 μg/mL. After 48 h of incubation, IFN-γ production was determined by IFN-γ CBA assay (BD Bioscience). GS-H2/4-1BB cells were incubated with 0.001–1 μg/mL of LYGN6051 anti-human 4-1BB Ab in the presence of single human FcγR-expressing cells. Two days later, IL-8 secretion in the supernatant was determined with human IL-8 HTRF cytokine assay kit (Cisbio) according to the manual.

### Detection of endotoxin in mAb preparation

Endotoxin was measured by the limulus amebocyte lysate assay (Cambrex inc., MD). For all mAb preparations, the amount of endotoxin was determined to be < 0.2 E.U./mg mAb.

### Liver infiltrated leukocytes preparation

Liver tissue was chopped by scissors and digested with 0.2 mg/mL of Liberase (Roche) and 0.25 mg/mL of DNase I (Sigma) at 37 °C for 30 min. The reaction was terminated by adding FBS and EDTA to a final concentration of 10% and 10 mM. The digested suspension was further purified by 70% and 37% Percoll gradient centrifugation. The interface layer was collected for further analysis by flow cytometry.

### Generation of bone marrow chimeras

WT mice were lethally irradiated with a single dose of 950 rad. The next day, irradiated mice were adoptively transferred with 2–3 × 10^6^ h4-1BB KI donor bone marrow cells. Mice were maintained on sulfamethoxazole and trimethoprim (Bactrim) antibiotics diluted in drinking water for 4 weeks after reconstitution. Mice were used for tumor or liver toxicity experiments ~4 weeks post reconstitution.

### Hepatotoxicity measurement

To study the hepatotoxic effects, mice were treated with 200 μg of indicated anti-4-1BB Abs i.p. weekly for 3 weeks. Seven days after the last antibody injection, alanine aminotransferase (ALT) levels in the blood were analyzed using the ALT/GPT Enzymatic Assay Kit (BioSino, Beijing, China) following the manufacturer’s instructions. Fourteen days after the last antibody injection, liver pathology was assessed by H&E and IHC staining by Servicebio (Wuhan, China).

### In vitro co-stimulation assay

For total mouse splenocyte-based or human PBMC-based co-stimulation assays: unpurified cells (4 × 10^5^) were stimulated with 0.1 μg/mL of anti-CD3 and 2 μg/mL of indicated anti-4-1BB Abs. Two days later, supernatants were collected and IFN-γ was measured by CBA. For the purified T cell-based co-stimulation assay, purified T cells (2 × 10^5^) were stimulated with 0.1 μg/mL of anti-CD3 and 2 μg/mL of indicated anti-4-1BB Abs when single FcγR-expressing cells (2 × 10^4^) are present. Two days later, the supernatants were collected and IFN-γ was measured by CBA assay.

### Flow cytometric analysis

Single cell suspensions of cells were incubated with anti-CD16/32 (anti-FcγRII/III, clone 2.4G2) for 10 min and then subsequently stained with conjugated Abs. The purchase and dilution information of all fluorescently labeled monoclonal Abs is listed as the following: αCD4-FITC (#100406,1:200), αCD8a-AF700 (#100730, 1:200), αCD69-APC (#104513, 1:200), αCD44-PE (#103007, 1:200), αIFN-γ-APC(#505810, 1:200), αCD64-APC(#139305, 1:200), αCD16/32-PE/Cy7(#101317, 1:200), αCD16.2-FITC(#149513, 1:200), and α4-1BB-APC(#106109, 1:200) from Biolegend; αCD32b-APC(#17-0321-80, 1:200) from eBioscience. Samples were analyzed on a Cytoflex (Beckman Coulter), and data were analyzed with FlowJo software (TreeStar, Inc.).

### Statistical analysis

Mean values were compared using an unpaired Student’s two-tailed *t*-test. Error bars represent SD or SEM. Statistically significant differences, not significant, *p* < 0.05, and *p* < 0.01 are noted with ns, * and **, respectively.

### Reporting summary

Further information on research design is available in the Nature Research Reporting Summary linked to this article.

## Supplementary information

Supplementary Information

Reporting Summary

Source Data

## Data Availability

Supporting data of this study are available from the corresponding author on reasonable request. Source data for graphs and tables presented in this manuscript are provided as a Source Data file. LVGN6051 is under clinical trials and its availability may be restricted. LVGN6051 may be available upon request after signing a Material Transfer Agreement with Lyvgen Biopharma.
