# Supplementary material for: Optimization of 4-1BB antibody for cancer immunotherapy by balancing agonistic strength with FcγR affinity
Source: Nat Commun. 2019 May 20;10:2141. doi: 10.1038/s41467-019-10088-1 (PMC6526162; doi:10.1038/s41467-019-10088-1)
Supplement: Supplementary file 1 — Supplementary Information [file 41467_2019_10088_MOESM1_ESM.pdf]

Supplemental Information

Optimization of 4-1BB antibody for cancer immunotherapy by balancing agonistic strength with FcγR affinity

Qi et al.

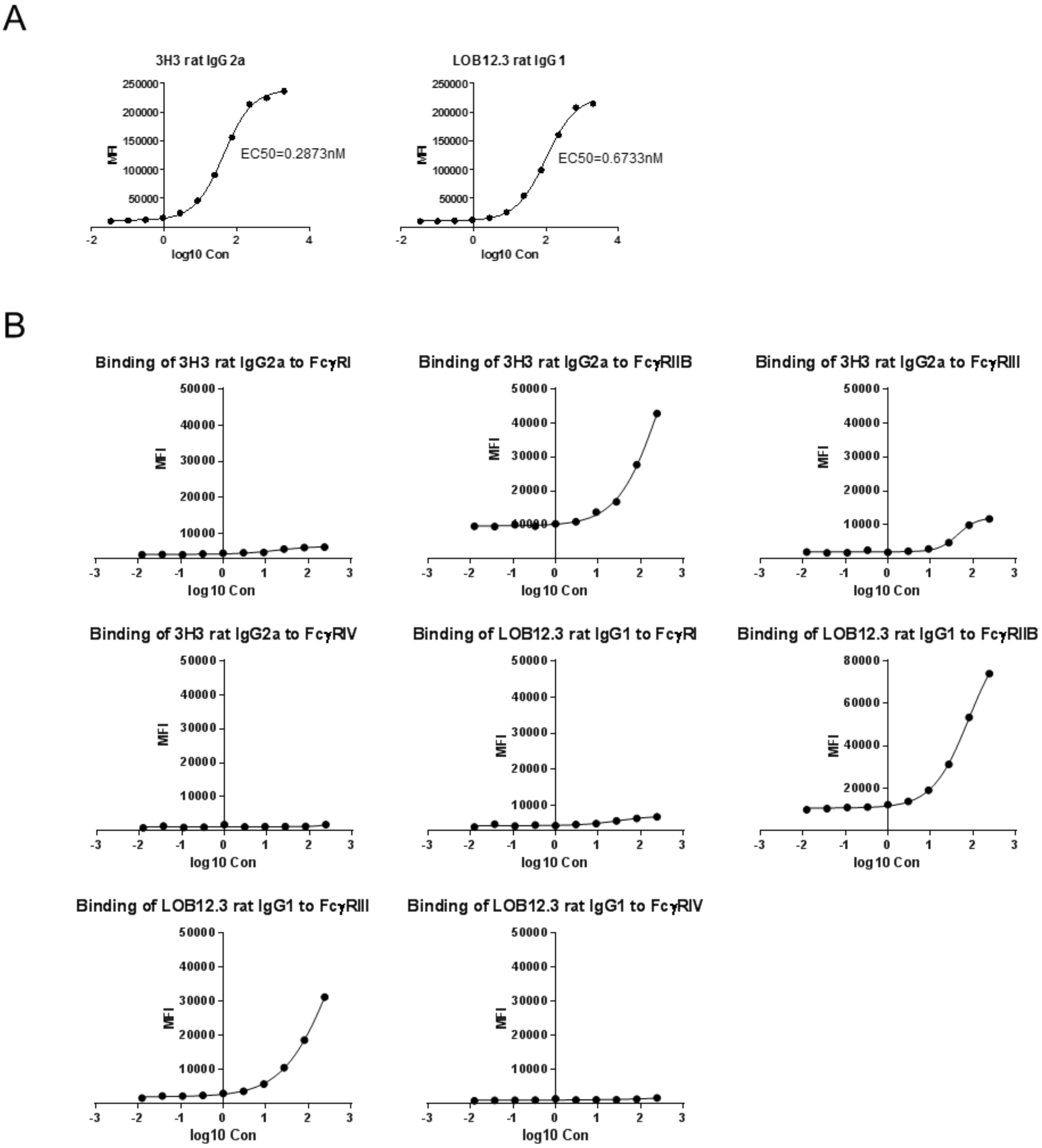

Supplementary Fig. 1. (A) Binding affinity of different anti-4-1BB Abs. 293-m-4-1BB cells were stained with different concentration of 3H3 and LOB12.3 Abs and followed by anti-rat Fc-AF647 secondary Ab. The EC50 was calculated by GraphPad software. (B) Mouse FcγRs binding profile of rat IgG1 and rat IgG2a. B16-OVA-mFcγRI, B16-OVA-mFcγRIIB, B16-OVA-mFcγRIII or B16-OVA-mFcγRIV were stained with different concentration of 3H3 ratIgG2a or LOB12.3 ratIgG1 and analyzed by flow cytometry.

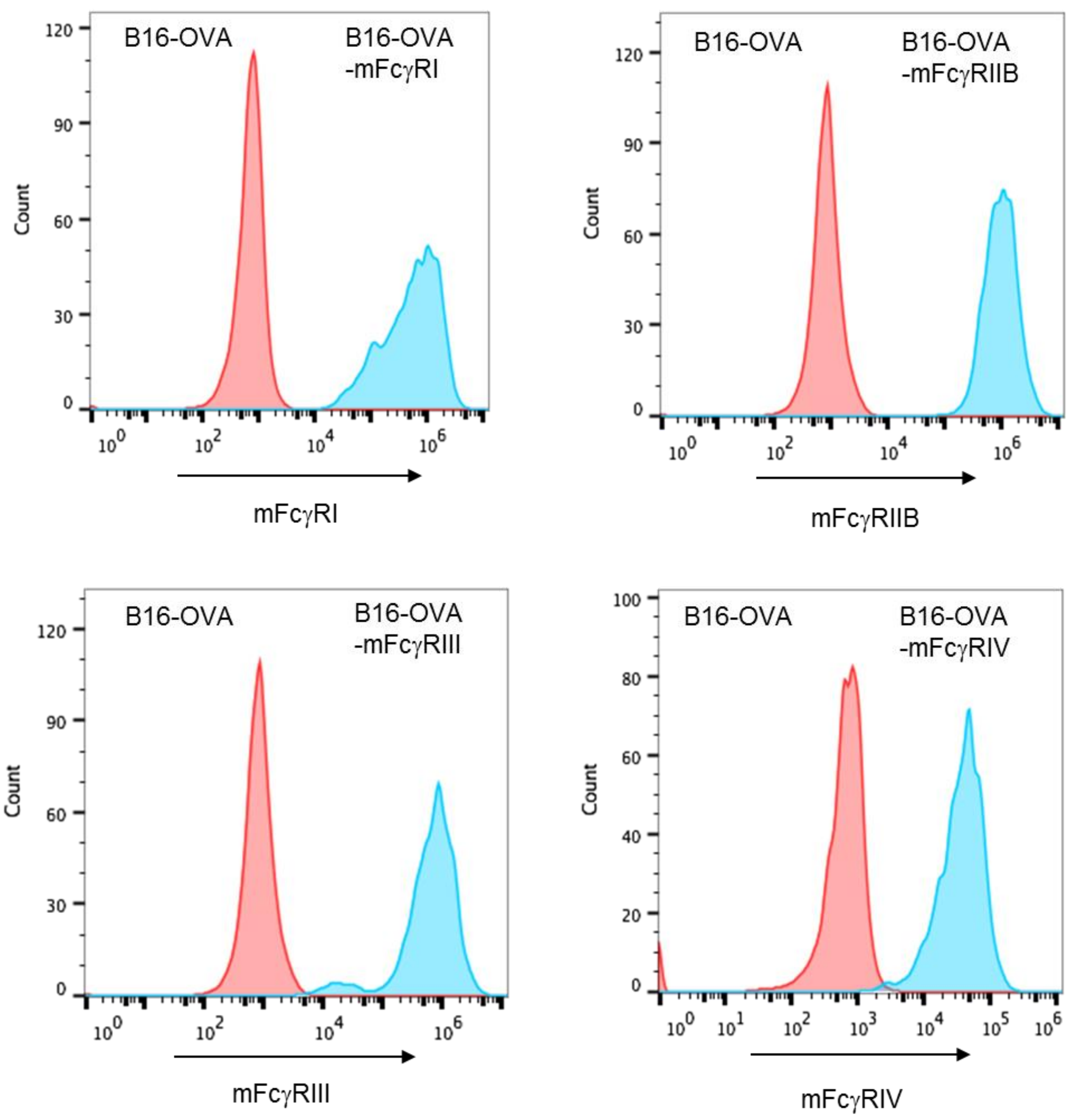

Supplementary Fig. 2. Mono-mouse-FcγR expressing cell line. Expression of mouse FcγR in B16-OVA stable cell lines was analyzed by flow cytometry.

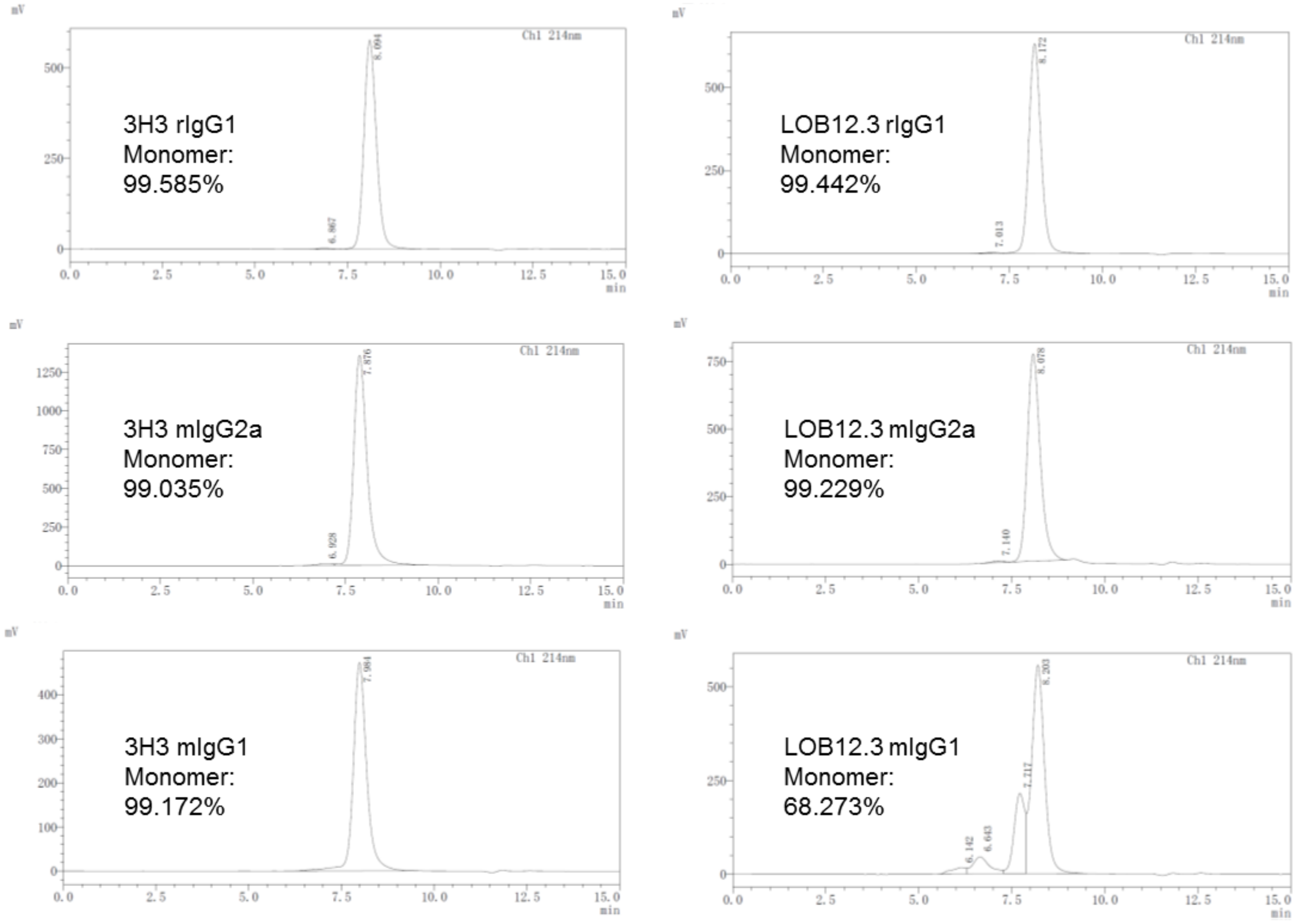

Supplementary Fig. 3. Size exclusion chromatography analysis of indicated anti-4-1BB Abs.

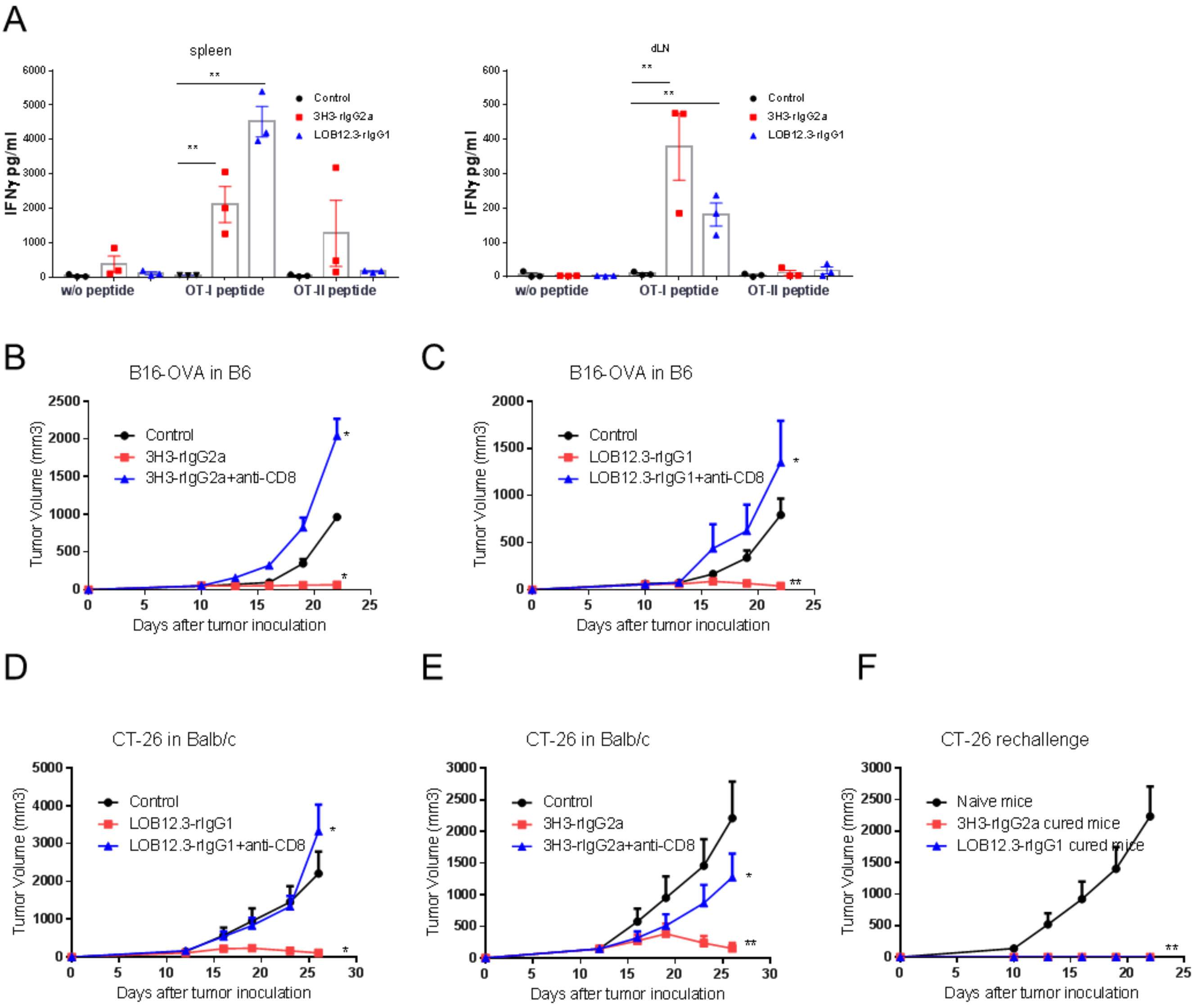

Supplementary Fig. 4. Anti-4-1BB Abs induced tumor regression is dependent on tumor specific CTL responses. (A) WT B6 mice were injected subcutaneously with 1×10^6 B16-OVA cells, then 200 μg of anti-4-1BB Abs or control IgG was administered on day 9. Seven days after the treatment, splenocytes and dLN cells were collected and IFN-γ CBA assay was performed after OT-I and OT-II peptide restimulation. (B-C) WT B6 mice (n=3-5/group) were injected subcutaneously with 1×10^6 B16-OVA cells, then 200 μg of indicated control IgG or anti-4-1BB Abs were administered on days 9, 16 and 23. Two hundred μg of anti-CD8 antibodies were administrated on days 9 and 16. Tumor growth was measured and compared twice a week. (D-E) Similar as in (B-C) (n=4-5/group), CD8 depletion assay was performed in CT-26 bearing Balb/c mice. (F) Naïve and tumor free mice from (D-E) (n=6/group) were rechallenged with 5×10^6 CT-26 cells and tumor growth was recorded and compared twice a week. Mean + SEM are shown.* $p < 0.05$, **$p<0.01$ compared with control group.

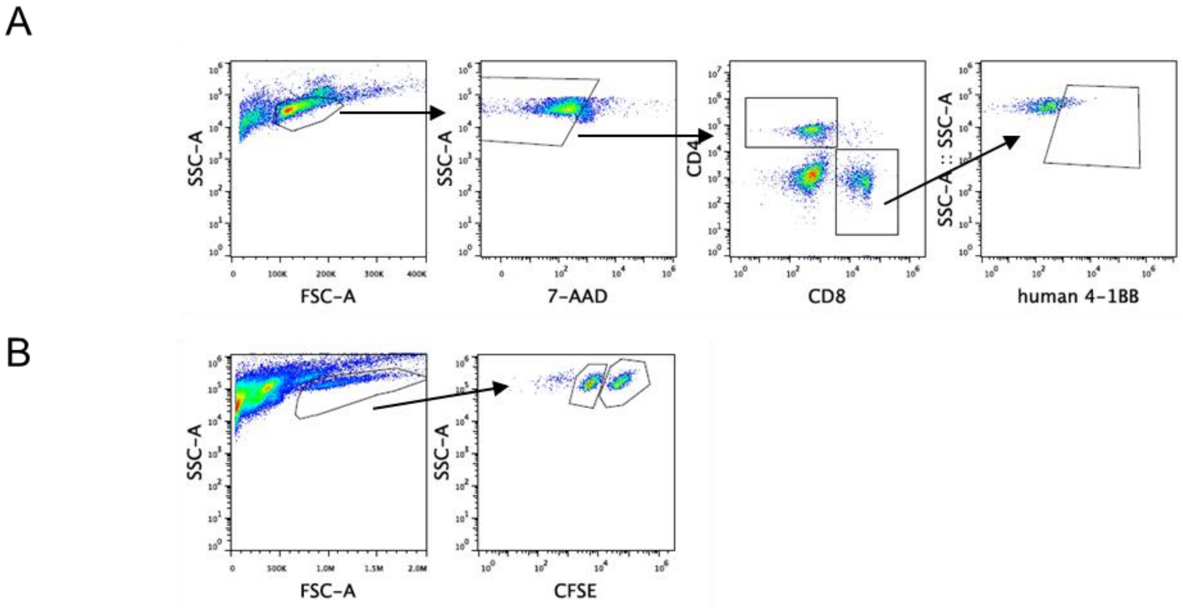

Supplementary Fig. 5. Flow cytometry sequential gating strategies of Fig. 3H, 3I and 3J. (A) Splenocytes from treated mice were analyzed by flow cytometry. Live splenocytes (7-AAD$^-$), CD4$^+$ T cell (CD4$^+$CD8$^-$), CD8$^+$ T cell (CD4$^-$CD8$^+$), 4-1BB$^+$ CD8 T cell (4-1BB$^+$ on CD8 T cell). (B) Target cells are distinguished by CFSE expression levels. EL4-4-1BB$^+$ (CFSE^{high}) and EL4-4-1BB$^-$ (CFSE^{low}).

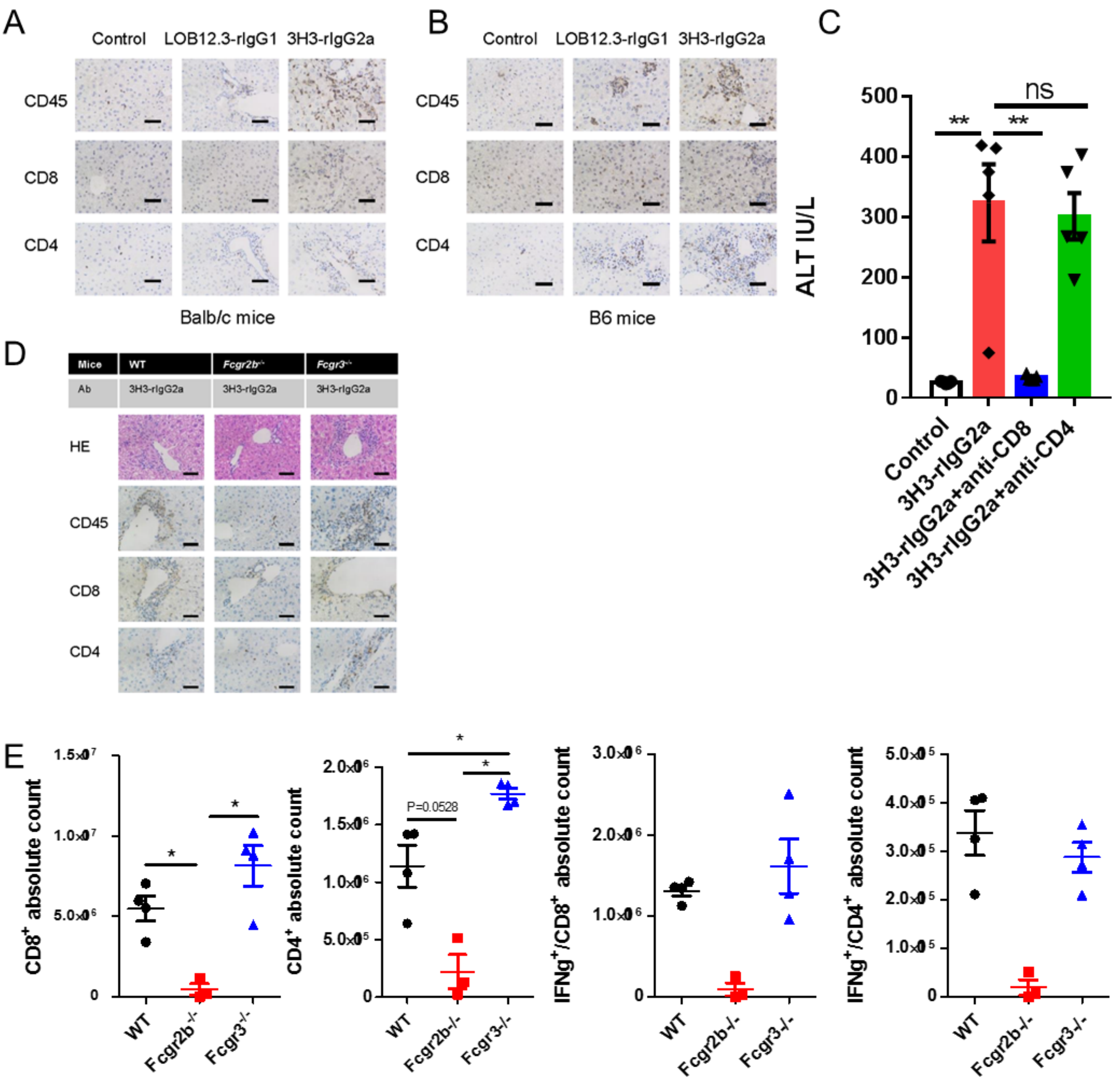

Supplementary Fig. 6. Liver immune cell infiltration after different anti-4-1BB Abs treatment. (A) Naïve Balb/c or (B) B6 mice were treated with 200μg of anti-4-1BB Ab (3H3 or LOB12.3) or control IgG on days 0, 7 and 14, Liver tissue was collected for indicated IHC staining on day 28. Liver immune cell infiltration after anti-4-1BB Ab (3H3) treatment in *Fcgr2b*$^{-/-}$ and *Fcgr3*$^{-/-}$ mice. (C) CD4^{+} T cells are not required for 3H3-rIgG2a induced liver toxicity. WT Balb/c mice (n=5/group) were treated with 3H3-rIgG2a on days 0, 7 and 14. Two hundred μg of anti-CD8 or CD4 antibodies were administrated on days 0 and 7. The ALT level in serum was analyzed on day 21. (D) WT, *Fcgr2b*$^{-/-}$ or *Fcgr3*$^{-/-}$ mice were administered on days 0, 7 and 14. Liver tissue was collected for H&E and indicated IHC staining on day 28. (E) Same as in (D)(n=3-4/group), liver infiltration cells were analyzed by flow cytometry. Scale bar: 50 μm. Mean + SEM are shown.* $p < 0.05$ ， **$p<0.01$ compared with control group.

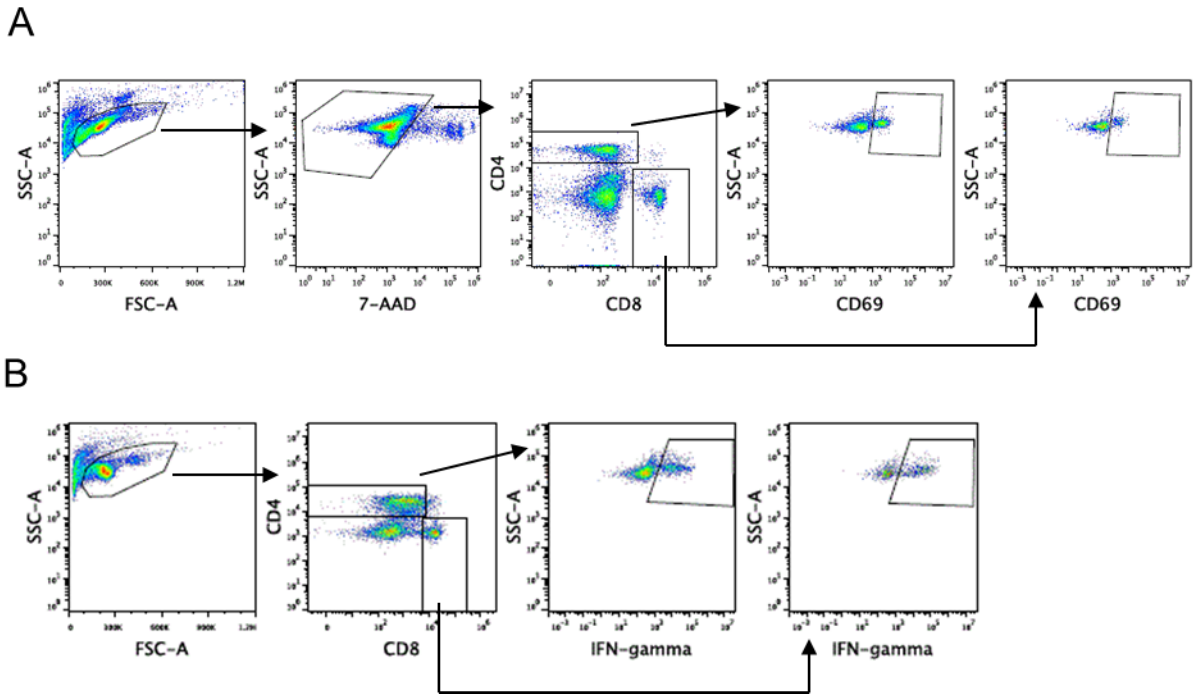

Supplementary Figure. 7. Flow cytometry sequential gating strategies of Fig. 4A and supplementary Fig. 6E. (A) Gating strategies to analysis live lymphocyte(7-AAD$^-$), CD4$^+$ T cell (CD4$^+$CD8$^-$), CD8$^+$ T cell (CD4$^-$CD8$^+$), activated CD4$^+$ T cell (CD69$^+$ on CD4$^+$ T cell), and activated CD8$^+$ T cell (CD69$^+$ on CD8$^+$ T cell). (B) CD4$^+$ T cell (CD4$^+$CD8$^-$), CD8$^+$ T cell (CD4$^-$CD8$^+$), IFN-γ^+CD4$^+$T cell (IFN-γ^+ on CD4$^+$ T cell), and IFN-γ^+ CD8$^+$ T cell (IFN-γ^+ on CD8$^+$ T cell).

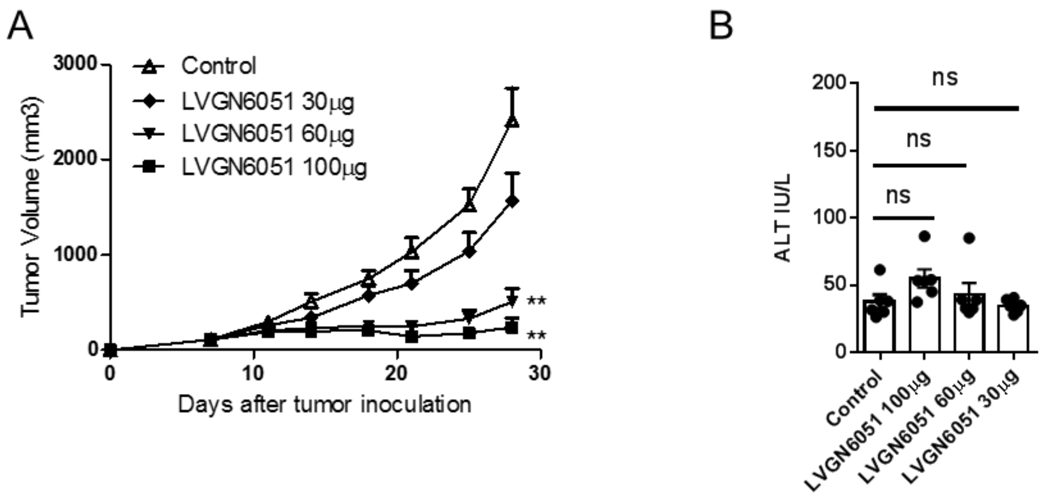

Supplementary Fig. 8. The anti-tumor efficacy and liver toxicity profile of LVGN6051. (A) Tumor bearing human 4-1BB KI mice were treated with 30, 60 or 100μg of LVGN6051. Tumor growth was measured twice a week. (B) Twenty one days after treatment, serum ALT levels in (A) were measured.
