# Supplementary material for: Optimization of 4-1BB antibody for cancer immunotherapy by balancing agonistic strength with FcγR affinity
Source: Nat Commun. 2019 May 20;10:2141. doi: 10.1038/s41467-019-10088-1 (PMC6526162; doi:10.1038/s41467-019-10088-1)
Supplement: Supplementary file 2 — Reporting Summary [file 41467_2019_10088_MOESM2_ESM.pdf]

nature research

Corresponding author(s): Xuanming Yang

Reporting Summary

Nature Research wishes to improve the reproducibility of the work that we publish. This form provides structure for consistency and transparency in reporting. For further information on Nature Research policies, see Authors & Referees and the Editorial Policy Checklist.

Statistical parameters

When statistical analyses are reported, confirm that the following items are present in the relevant location (e.g. figure legend, table legend, main text, or Methods section).

n/a	Confirmed	
☐	☒	The exact sample size (*n*) for each experimental group/condition, given as a discrete number and unit of measurement
☒	☐	An indication of whether measurements were taken from distinct samples or whether the same sample was measured repeatedly
☐	☒	The statistical test(s) used AND whether they are one- or two-sided<br>*Only common tests should be described solely by name; describe more complex techniques in the Methods section.*
☒	☐	A description of all covariates tested
☒	☐	A description of any assumptions or corrections, such as tests of normality and adjustment for multiple comparisons
☒	☐	A full description of the statistics including central tendency (e.g. means) or other basic estimates (e.g. regression coefficient) AND variation (e.g. standard deviation) or associated estimates of uncertainty (e.g. confidence intervals)
☐	☒	For null hypothesis testing, the test statistic (e.g. *F*, *t*, *r*) with confidence intervals, effect sizes, degrees of freedom and *P* value noted<br>*Give P values as exact values whenever suitable.*
☒	☐	For Bayesian analysis, information on the choice of priors and Markov chain Monte Carlo settings
☒	☐	For hierarchical and complex designs, identification of the appropriate level for tests and full reporting of outcomes
☒	☐	Estimates of effect sizes (e.g. Cohen's *d*, Pearson's *r*), indicating how they were calculated
☐	☒	Clearly defined error bars<br>*State explicitly what error bars represent (e.g. SD, SE, CI)*

Our web collection on statistics for biologists may be useful.

Software and code

Policy information about availability of computer code

Data collection	N/A
Data analysis	N/A

For manuscripts utilizing custom algorithms or software that are central to the research but not yet described in published literature, software must be made available to editors/reviewers upon request. We strongly encourage code deposition in a community repository (e.g. GitHub). See the Nature Research guidelines for submitting code & software for further information.

Data

Policy information about availability of data

All manuscripts must include a data availability statement. This statement should provide the following information, where applicable:

- Accession codes, unique identifiers, or web links for publicly available datasets
- A list of figures that have associated raw data
- A description of any restrictions on data availability

The authors declare that all the data supporting the findings of this study are available from the authors on reasonable request.

April 2018

Field-specific reporting

Please select the best fit for your research. If you are not sure, read the appropriate sections before making your selection.

☒ Life sciences ☐ Behavioural & social sciences ☐ Ecological, evolutionary & environmental sciences

For a reference copy of the document with all sections, see nature.com/authors/policies/ReportingSummary-flat.pdf

Life sciences study design

All studies must disclose on these points even when the disclosure is negative.

Sample size	We did not predetermine sample size. All experiments were performed multiple times. If results were reproducible, the conclusions were consider valid.
Data exclusions	No data were excluded.
Replication	All attempts at replication were successful.
Randomization	Samples were not randomized. For tumor experiments, mice were allocated to different groups with even initial tumor size.
Blinding	The investigators were not blinded to group allocation during data collection and analysis.

Reporting for specific materials, systems and methods

Materials & experimental systems

n/a	Involved in the study	
☐	☒	Unique biological materials
☐	☒	Antibodies
☐	☒	Eukaryotic cell lines
☒	☐	Palaeontology
☐	☒	Animals and other organisms
☒	☐	Human research participants

Methods

n/a	Involved in the study	
☒	☐	ChIP-seq
☐	☒	Flow cytometry
☒	☐	MRI-based neuroimaging

Unique biological materials

Policy information about availability of materials

Obtaining unique materials	LVGN6051 is under clinical trials and its availabilty may be restricted. LVGN6051 may be available upon request after signing a Material Transfer Agreement with Lyvgen Biopharma.

Antibodies

Antibodies used	anti-CD4-FITC(#100406,1:200), anti-CD8a-AF700(#100730, 1:200), anti-CD69-APC(#104513, 1:200), anti-CD44-PE(#103007, 1:200), anti-IFN-g APC(#505810, 1:200), anti-CD64-APC(#139305, 1:200), anti-CD16/32-PE/Cy7(#101317, 1:200), anti-CD16.2-FITC(#149513, 1:200) and anti-4-1BB-APC(#106109, 1:200) from Biolegend; anti-CD32b-APC(#17-0321-80, 1:200) from Ebioscience. Mouse anti-rat IgG (Fab)2 -AF647(#212-605-106, 1:1000) from Jackson Immuno research.
Validation	*Describe the validation of each primary antibody for the species and application, noting any validation statements on the manufacturer's website, relevant citations, antibody profiles in online databases, or data provided in the manuscript.*

Eukaryotic cell lines

Policy information about cell lines

Cell line source(s)	B16-OVA was kindly provided by Hans Schreiber (The University of Chicago). 3T3 and CT-26 were kindly provided by Stem Cell Bank, Chinese Academy of Sciences （Shanghai, China） .

Authentication | None of the cell lines used were authenticated.

Mycoplasma contamination | All cell lines are tested negative for mycoplasma.

Commonly misidentified lines (See ICLAC register) | *Name any commonly misidentified cell lines used in the study and provide a rationale for their use.*

Animals and other organisms

Policy information about studies involving animals; ARRIVE guidelines recommended for reporting animal research

Laboratory animals | C57BL/6J and Balb/c mice were purchased from Beijing Vital River Laboratory Animal Technology Co., Ltd. (Beijing, China). Fcgr2b-/- and Fcgr3-/- mice were purchased from JAX. Human 4-1BB knock-in mice were purchased from Biocytogen, Inc (Beijing, China). Both male and female mice were used for the study.

Wild animals | The study did not involve wild animals.

Field-collected samples | The study did not involve samples collected from the field.

Flow Cytometry

Plots

Confirm that:

☒ The axis labels state the marker and fluorochrome used (e.g. CD4-FITC).

☒ The axis scales are clearly visible. Include numbers along axes only for bottom left plot of group (a 'group' is an analysis of identical markers).

☒ All plots are contour plots with outliers or pseudocolor plots.

☒ A numerical value for number of cells or percentage (with statistics) is provided.

Methodology

Sample preparation | Liver tissue and tumor tissue was chopped by scissors and digested with 0.2 mg/ml of Liberase (Roche) and 0.25 mg/ml of DNase I (Sigma) at 37 degree for 30 minutes. The reaction was terminated by adding FBS and EDTA to a final concentration of 10% and 10mM. The digested suspension was further purified by 70% and 37% Percoll gradient centrifugation. The interface layer was collected for further analysis by flow cytometry.

Instrument | Cytoflex S (Beckman Coulter)

Software | Flowjo

Cell population abundance | N/A

Gating strategy | Cells were firstly gated based on FSC/SSC. Then 7AAD- cells were gated as live cells for further analysis.

☒ Tick this box to confirm that a figure exemplifying the gating strategy is provided in the Supplementary Information.
